# The Clinical Application of Gel-Based Composite Scaffolds in Rotator Cuff Repair

**DOI:** 10.3390/gels11010002

**Published:** 2024-12-24

**Authors:** Shebin Tharakan, Michael Hadjiargyrou, Azhar Ilyas

**Affiliations:** 1 Bio-Nanotechnology and Biomaterials (BNB) Laboratory, New York Institute of Technology, Old Westbury, NY 11568, USA; 2 College of Osteopathic Medicine, New York Institute of Technology, Old Westbury, NY 11568, USA; 3 Department of Biological & Chemical Sciences, New York Institute of Technology, Old Westbury, NY 11568, USA; mhadji@nyit.edu; 4 Department Electrical and Computer Engineering, New York Institute of Technology, Old Westbury, NY 11568, USA

**Keywords:** rotator cuff, gel-based composite scaffolds, grafts, clinical outcomes, augmentation, orthopedic surgery

## Abstract

Rotator cuff tears are a common injury that can be treated with or without surgical intervention. Gel-based scaffolds have gained significant attention in the field of tissue engineering, particularly for applications like rotator cuff repair. Scaffolds can be biological, synthetic, or a mixture of both materials. Collagen, a primary constituent of the extracellular matrix (ECM) in musculoskeletal tissues, is one of the most widely used materials for gel-based scaffolds in rotator cuff repair, but other ECM-based and synthetic-based composite scaffolds have also been utilized. These composite scaffolds can be engineered to mimic the biomechanical and biological properties of natural tissues, supporting the healing process and promoting regeneration. Various clinical studies examined the effectiveness of these composite scaffolds with collagen, ECM and synthetic polymers and provided outstanding results with remarkable improvements in range of motion (ROM), strength, and pain. This review explores the material composition, manufacturing process and material properties of gel-based composite scaffolds as well as their clinical outcomes for the treatment of rotator cuff injuries.

## 1. Introduction

The human shoulder is very complex and dynamic with multiple anatomical structures that stabilize the glenohumeral synovial joint. Compared to the acetabulum, the glenoid fossa is shallow conferring greater mobility in exchange for stability making the shoulder prone to luxation [1]. The four muscles stabilizing the glenohumeral joint are collectively known as the rotator cuff. The rotator cuff consists of the subscapularis, infraspinatus, supraspinatus, and teres minor muscles anchoring the humeral head to the glenoid cavity [2]. In addition to the rotator cuff, accessory structures such as the bursae and the fibrocartilage labrum enhance joint stability [3]. Injuries of the shoulder may arise due to overuse, sports injuries, or potential genetic predisposition which result in muscle impingement or decreased range of motion (ROM) [4,5,6]. Age also plays a factor in decreased ROM as a recent study in a community-based cohort indicated that flexion, abduction, and external rotation tend to decrease with age [7].

A rotator cuff tear entails damage to the tendons surrounding the glenohumeral joint. The most common tear involves the supraspinatus tendon which presents with a decreased ROM, a positive Jobe’s test, and a subacromial grind test [8,9,10]. Two types of tears are a partial-thickness tear (PTT) or a full-thickness tear (FTT). PTTs are incomplete tears that arise due to trauma but can propagate into an FTT over time developing into a cuff tear arthropathy. FTTs involve tendon discontinuance due to trauma which does not heal without aid and often require surgical intervention [11]. Currently, rotator cuff tears are prevalent in athletes and older individuals. Shoulder overuse by overhead motion as seen in athletes pose the threat of impingement and rotator cuff pathology. In particular, athletes older than 40 years are at significant risk for rotator cuff tears after an anterior dislocation [12]. Previous studies have determined rotator cuff tears (asymptomatic and symptomatic) are in tandem with age affecting individuals 60 years and older [13,14]. Bilateral cuff tears correlate with age as one study indicated a 50% likelihood of tearing after the age of 66 [15].

Clinical management of rotator cuff tears may be nonoperative or operative dependent upon the pathology. Conservative measures for management include NSAID treatments, muscle massages, and physiotherapy for the initial treatment of PTTs [16]. Operative measures for PTT repair include surgical debridement or arthroscopic tendon repair [17]. Subacromial decompression may also be used for Ellman Grade I or II without the need for rotator cuff repair for long-term outcomes [18]. FTTs may be managed with analgesics but operative procedures are necessary to prevent further degeneration [19]. Surgical intervention for FTTs showed to have a positive long-term response indicating that 80% of rotator cuff repairs were excellent or satisfactory after an average of 13 years [20]. Traditional repair methods, such as suturing, may fail to restore the normal structure and function of the tendon, leading to re-tears. Surgical management also showed promising results with the use of biological or synthetic 3D tissue engineered scaffolds in the repair of rotator cuff tears [21]. This has prompted the development of advanced biomaterials like gel-based composite scaffolds to improve tissue regeneration and healing. Below, we describe the various types of gel-based scaffolds used for the repair of rotator cuff tears.

## 2. Material Composition, Manufacturing Process and Properties of Gel-Based Composite Scaffolds

### 2.1. Materials for Gel-Based Composite Scaffolds

Gel-based composite scaffolds for rotator cuff repair are an evolving area of research, with promising potential to improve healing outcomes. The combination of natural and synthetic materials (composite scaffolds) offers a balance between biological compatibility and mechanical strength [22]. For example, collagen, hyaluronic acid, and fibrin are highly beneficial for promoting cellular interactions and tissue regeneration, while Poly(lactic-co-glycolic acid) (PLGA) and Polyethylene glycol (PEG) provide superior mechanical strength [23,24]. Table 1 provides an overview of the key gel-based composite scaffold materials used for rotator cuff repair, comparing their material and biological properties.

### 2.2. Manufacturing Processes of Gel-Based Composite Scaffolds

The manufacturing processes and technological innovations of gel-based composite scaffolds are critical for their successful application in tissue engineering, particularly for complex repairs like rotator cuff injuries. These processes aim to produce scaffolds with the ideal mechanical properties, biocompatibility, and degradation rates, while promoting cell adhesion, migration, and tissue regeneration. Table 2 provides an overview of each manufacturing process, its technological innovations, and how they compare in terms of benefits, challenges, and applications.

### 2.3. Mechanical and Biological Propoerties of Gel-Based Composite Scaffolds

Gel-based composite scaffolds for rotator cuff repair integrate exceptional mechanical and biological properties, making them highly effective for tissue regeneration. Biologically, these scaffolds offer high biocompatibility, supporting cell adhesion, proliferation, and migration [21]. Their porous structure allows for nutrient and oxygen diffusion, cell migration, fostering angiogenesis and new tissue formation. Bioactive molecules such as growth factors (e.g., TGF-β, BMPs) and other types of therapeutic agents can be integrated into the scaffold to promote tendon-bone integration and control inflammation [50,51]. The controlled degradability of the materials ensures the scaffold provides temporary support while gradually being replaced by native ECM without generating harmful toxic by-products that can elicit a local or even a systemic immune response.

Mechanically, these scaffolds are engineered to match the elasticity and tensile strength of native tendons, providing resistance to physiological loads [52]. Their stiffness gradient [53], and bone interface, enable efficient load transfer and reduced mechanical discrepancies [54]. Fatigue resistance and durability, enhanced through the incorporation of various types of nanomaterials, ensure the scaffold’s structural integrity during repetitive movements [55,56]. These combined properties make gel-based composite scaffolds a promising solution for improving outcomes in clinical treatment of rotator cuff repair. Table 3 provides an overview of the mechanical properties of gel-based composite scaffolds.

## 3. Scaffold Augmentations

**Figure 1:**
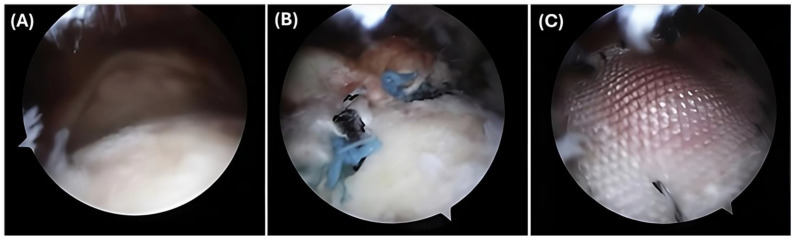
Right shoulder observed from the lateral portal. (**A**) Demonstrates a large tear at the level of the glenoid. (**B**) Rotator cuff repair prior to the addition of the synthetic PLLA scaffold. (**C**) Placement of the scaffold with medial sutures and anchors. Reprinted with permissions from [66].

As aforementioned, gel-based and synthetic composite scaffolds can be biodegradable and encourage cell proliferation for tendon or muscle repair [60]. They can consist of different biomaterials forming composites or one material either biological or synthetic [61,62]. The bioengineered constructs may contain collagen, ECM, decellularized ECM, human/porcine dermis, or biosynthetic material which can be implanted into the rotator cuff in a process known as scaffold augmentation [63]. Figure 1 demonstrates one example using a synthetic Poly-L-lactic acid (PLLA)-based scaffold augmentation for rotator cuff repair. This type of scaffold augmentation for shoulder treatment has been tested in different in vivo models which showed positive results [64,65].

**Figure 2:**
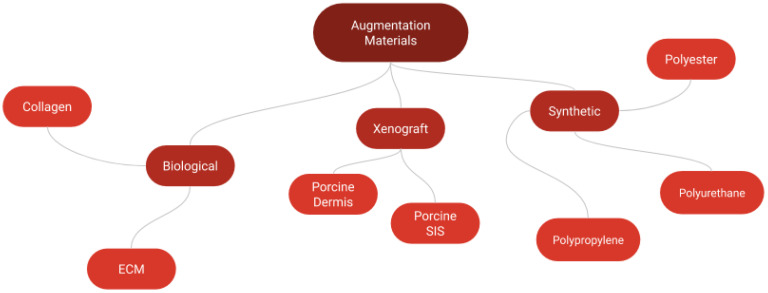
An example of some biological and synthetic materials that can constitute composite scaffold for shoulder augmentation.

Gel-based scaffolds typically consist of biocompatible and biodegradable materials that mimic the natural ECM, providing a 3D structure for cellular growth and tissue development. They are typically designed to be injectable or moldable, allowing them to conform to the shape of the damaged area. These scaffolds support cell attachment, proliferation, and differentiation, and can be loaded with bioactive factors to enhance healing. These scaffolds can be inserted and fixed arthroscopically onto the rotator cuff [67]. Collagen, ECM, cells, and growth factors can constitute the composite scaffold to enhance regeneration and recovery [68,69,70]. Figure 2 provides an example of some materials that can constitute composite scaffolds. Previous in vivo studies conducted on rats, sheep, and canine models with synthetic or biological scaffolds have demonstrated positive outcomes for augmentation. Tenocyte growth was observed with little immune or inflammatory response present and the scaffolds were also biodegradable [71,72,73,74]. Unfortunately, that does not necessitate the same outcome clinically. Scaffolds have been used clinically for spinal fusion, fractures and defects, sinus augmentation, hand surgery, and cranioplasty with varying clinical outcomes [75]. In the case of shoulder augmentation, different clinical outcomes have been reported from previous scaffold evaluations [68,76,77] and can be grouped into three major categories: (i) Collagen-based (ii) ECM-based and (iii) Synthetic-based augmentation. A summary of several studies dealing with scaffold augmentation is represented in Table 4 below.

### 3.1. Collagen-Based Augmentation

Collagen scaffolds have been used pre-clinically and clinically, and demonstrated tendon regeneration and remodeling [74,98]. Bokor et al. [79] utilized porous type I collagen scaffolds in regenerating PTTs of the supraspinatus tendon. The study involved 13 patients evaluated by MRI over 2 years to monitor tendon regeneration. The scaffolds were placed on the bursal side arthroscopically and were observed to increase tendon thickness throughout the study. A total of 92% of the patients experienced a positive result in decreasing tear size. Tears regressed from high-grade to low-grade or none as the scaffold demonstrated potent healing capabilities in reducing the defect size. The collagen-based scaffold provided inductive capabilities to assist in the formation of new connective tissue [74,79]. A 5-year follow-up on 11 patients from this study was conducted by the same authors. Tendon thickness decreased at the 5-year MRI but was better than the pre-operative thickness. Tendon integrity was established in 72.7% of the patients indicating no degenerative effects in this sample [78]. A separate but similar study conducted by Bokor et al. involved type I collagen scaffolds in FTT repair of the supraspinatus tendon. The study recruited nine patients, with 89% having satisfactory results after 24 months. Similar to the first study, the collagen-based scaffolds were placed on the bursal side and an MRI was used to evaluate patient status post-operatively. Tendon thickness increased for up to 6-months with new tendon growth indistinguishable from native tendon [80]. These results support the notion of utilizing collagen-based tissue engineered scaffolds for PTT and FTT repair, albeit in samples of 13 and 9 patients, respectively. Similarly, Thon et al. [81] arthroscopically implanted collagen scaffolds in patients with large or massive Cofield classified cuff tears. Ultrasound and MRI were used to determine the progress of tendon healing in 23 patients. A total of 96% of patients had tendon healing with a 6% failure rate due to two patients having either progressive arthritis or a lack of tendon healing. Overall tendon thickness and vascularity increased within 12 months as determined by imaging [81]. Schlegel et al. [82] recruited 33 patients for augmentation with a collagen patch for PTT repair. MRI assessment of the defects indicated that 70% of patients showed a decrease of at least 1 grade in tear severity 3 months post-augmentation. Six patients had their tears completely heal, with one individual having no reduction in tear size. Supraspinatus tendon thickness improved after 3 months and persisted until 12 months in 94% of the patients post-operatively. A 1-year follow-up indicated that all tear defect sizes decreased except for one [82]. A recent study conducted by Dai et al. used bovine-collagen-derived scaffolds from Smith and Nephew Regeneten for PTT repair in 30 patients with 24 available for follow-up. Tendon thickness increased post-operatively from 0.8 mm to 6.5 mm at the average follow-up. Additionally, the implant reduced strain on the supraspinatus tendon for bursal, articular, and intrasubstance tears due to its inductive abilities [83]. Furthermore, Bushnell et al. performed a larger study involving 115 patients with the Smith and Nephew Regeneten collagen scaffold for repairing FTTs and determined re-tear rates. Patients involved in the study had medium or large Cofield classified tears with the scaffold arthroscopically inserted. These collagen scaffolds were resorbed in 100% of patients at the 1-year follow up but a boundary was detected in 8.9% of patients at the 3-month checkup. This indicates good biocompatibility as the scaffold was able to encourage tissue growth [84]. There was a 16.5% re-tear rate within 1 year, including re-torn and failed to heal rotator cuffs which is lower than other reported studies [99,100,101,102,103]. Although these studies lack a control shoulder, improvements were observed with the use of scaffolds.

### 3.2. Extracellular Matrix-Based Augmentation

ECM-based hydrogels are typically injected into the injury site to fill gaps or defects, improving tendon healing by promoting cell infiltration, thus representing the predominant approach for augmentation [104,105]. Porcine products (acellular matrix or collagen membrane) were reported to have varying efficacy from older studies [85,106,107,108,109]. Iannotti et al. [85] studied 30 patients with large or massive supraspinatus or infraspinatus tendon tears with porcine small intestine submucosa (SIS) implants. A total of 89% of the cohort patients with large tears had fully healed, however, with massive tears only 24% were fully healed. Overall, 40% of the tears did not heal due to varying factors. There was no significant difference between groups and no clear clinical benefit was observed in the 30 shoulders evaluated [85]. A similar study was conducted by Bryant et al. [86] and included 62 randomized patients with porcine SIS augmentations. The primary outcome evaluated the success of the implants with 52.9% failure in the augmentation group and 65.4% failure in the control. Although the sample size was greater than Iannotti et al. [53], the risk ratio (0.81) was in favor of the augmentation group, but again, the results were not statistically significant between groups. Similar to Iannotti et al. [53], the authors concluded there was no apparent clinical benefit from using the SIS implant [86].

Castagna et al. [87] tested porcine dermis-derived collagen membranes in 35 patients for large or massive rotator cuff tears. The treatment group included 7 shoulders as compared to 13 in the control which experienced re-tears as determined by MRI evaluation at 24 months. Although tendon integrity was not fully restored by the implant, there was no evidence of tissue rejection due to the xenograft. As such, the authors concluded that the porcine dermis-derived collagen to be safe for human use [87]. A separate study evaluated a porcine dermal patch in 96 patients randomized into two arms. Avanzi et al. [88] followed up with 38 patients with augmentation and 30 without. Significant healing was present in the augmented group with a healing rate of 97.6% as compared to 59.6% in the control. Tendon thickness and footprint coverage were significantly higher in the augmented group throughout the 24 months. Density scores were similar with 87.3 and 88.4 for the augmented and control group, respectively. Additionally, the augmented group displayed a quicker tendon strength recovery. On the contrary, Maillot et al. [89] provide contradictory results with the use of porcine dermal patches. Thirty-two patients were placed into either an arthroscopic repair, patch, or debridement group and no difference was observed between the three groups. Recovery for anterior elevation and external rotation progressed slower as compared to the debridement and arthroscopic repair groups. The analysis of clinical outcomes indicated no superior benefit to utilizing the porcine dermal patch but is limited by the sample size of 11 patients [89].

**Figure 3:**
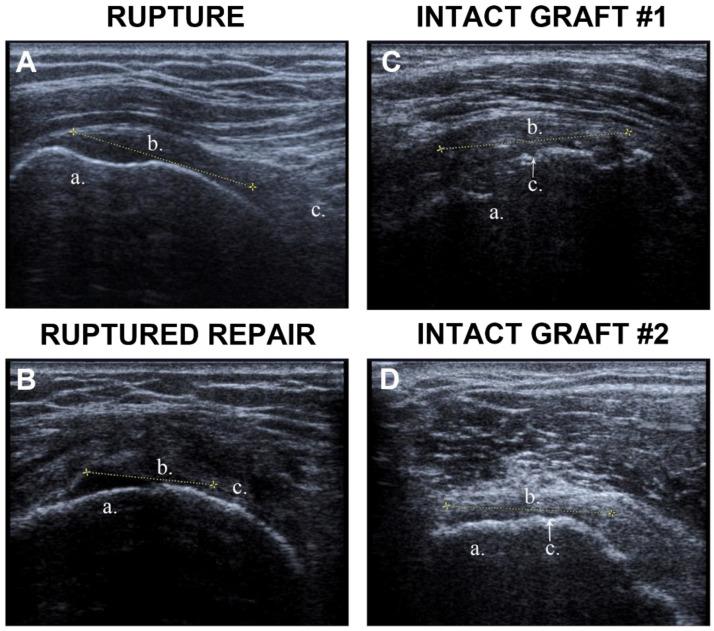
Ultrasound image of the rotator cuff tears and repairs from representative patients. (**A**) Rotator cuff tear. a. humeral head; b. defect; c. retracted cuff. (**B**) Ruptured repair. a. humeral head; b. defect; c. retracted cuff and graft. (**C**,**D**) Intact GraftJacket^®^ and cuff repair. a. humeral head; b. intact graft; c. suture. Adapted with permission from [91].

ECM based scaffolds using human acellular dermal allograft have also been tested. Gouk et al. [110] used a human acellular dermal allograft from Graft Jacket^®^ in eight patients as an interposition graft. MRI was used post-operatively and indicated that 14% of grafts were intact within 6 months. The other 86% had failed grafts within 6 months, consistent with previous reports that indicated failure as well [110]. Additionally, the grafts displayed integration deficits suggesting failed compatibility with the rotator cuff [90]. Alternatively, Johnson et al. [91] had positive outcomes with the same Graft Jacket^®^ patch on 14 shoulders. Ultrasound evaluation indicated intact grafts within 35 months with three grafts undergoing attenuation or heterogeneous change (Figure 3). Also, ROM was functional at a 2-year follow-up. The graft was viable at the final follow-up of 41 months and indicated healing of the rotator cuff as seen by shoulder functionality [91].

### 3.3. Synthetic-Based Augmentation

**Figure 4:**
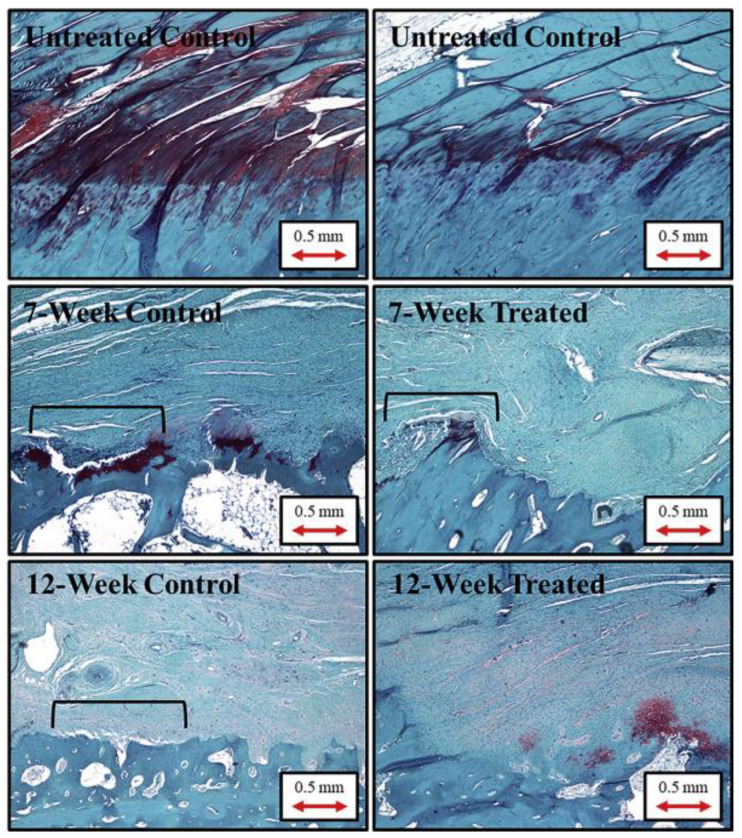
Histology specimens extracted from sheep infraspinatus tenocytes at the tendon-bone junction were stained with Safranin O, demonstrating healing with a PLGA anchored scaffold. Black brackets indicate interfaces where tissue and bone are not properly integrated. Magnification of 40×. Reprinted with permissions from [73].

Synthetic hydrogels are a group of synthetic polymers that can be photo-crosslinked, allowing for precise control over their physical properties, including stiffness and degradation rate. These scaffolds are versatile and can be tailored to provide the appropriate biomaterial properties of the target tissue. Synthetic composite scaffolds can incorporate polymers such as polyester, PLLA, polypropylene, polyacrylamide, Dacron, carbon, silicone, and nylon [111,112,113]. These synthetic composite materials are considered foreign bodies and are associated with complications due to poor biocompatibility [114]. Many studies were conducted on synthetic materials for rotator cuff repair with promising results due to their strong mechanical characteristics. Figure 4 shows tendon-bone junction extracts from sheep demonstrating proper healing in groups with PLGA scaffolds [73]. Encalada-Diaz et al. [92] used a non-resorbable polycarbonate polyurethane patch in 10 patients with FTTs to evaluate post-operative outcomes. Healing was present in 90% of patients as determined by MRI and ultrasound, while one patient experienced a re-tear. The low re-tear rate, progressive ROM, and high healing rate demonstrated efficacy but, the study was limited by the small sample size and sex (cohort of 10 females) [92]. A degradable polyurethane urea scaffold was employed by Petriccioli et al. [93] in 10 patients for the repair of the subscapularis. Ultrasound and MRI were also used to identify FTTs of the subscapularis in 1 patient who experienced a re-tear within 23 months. The remaining nine patients had intact repairs with all patients having greater tendon thickness in the repaired tendon compared to the healthy control shoulder. The scaffold provided a low re-tear rate in this small cohort [93]. Another study involved the use of a PLLA-based resorbable scaffold for massive rotator cuff tears in 18 patients. Results indicated an 83% success in repair rate 12 months post-operatively with long-term success at 78% by 42 months. Three repairs failed due to factors unrelated to the scaffold. The integrity of the rotator cuff was strengthened due to the stability of the scaffold [66]. Despite being the first clinical synthetic study, no control group was present, but the long-term results are indeed promising.

Polypropylene is another biosynthetic material that can be used in composite scaffolds. Ciampi et al. [94] separated 152 patients into three groups receiving a collagen patch, polypropylene patch, or no patch (control). Elevation and abduction strength at 36 months with the propylene patch was significantly higher than those with the collagen and control group, probably because propylene provides greater long-term strength since it is not resorbed, unlike collagen. The re-tear rate was also significantly lower in the propylene group, at 17% compared to 41% and 51% in the control and collagen group, respectively [94]. Similar results were reported by Vitali et al. [95] with the use of a propylene scaffold in 60 patients. Greater abduction strength and scapular plane elevation were observed in the propylene group. Re-tear rates were also lower at 15% in the propylene group as compared to 40% in the control group, demonstrating greater tendon integrity with the scaffold [95]. The improvements presented with the propylene scaffold shows clinical potential for reducing re-tears and increasing rotator cuff integrity. Polyester is another synthetic material that was tested by Smolen et al. [96] in 50 patients. There was a significant improvement in internal rotation within 22 months. Flexion, abduction, external rotation, and strength did improve post-operatively but were not significant. As for re-tears, 86% of patients had an intact repair with 14% experiencing a re-tear [96]. A recent study by Cowling et al. treated 29 patients with a polyester patch and 39 patients without. Comparable results with those of Smolen et al. [96] were observed since the augmentation with polyester improved shoulder functionality. However, the Goutallier classification of both groups showed little difference at 6 months. Although Smolen et al. [96] conducted a longer study, the authors acknowledged that 6 months may be too early to identify changes in the Goutallier classification [97]. Despite this, the results indicated beneficial short-term results with the polyester patch.

## 4. Patient Outcomes

The clinical implications for various types of gel-based scaffolds used for rotator cuff repair are described in Table 5.

### 4.1. Collagen-Based Scaffolds

The clinical assessment of Bokor et al. [39] in repairing supraspinatus PTTs indicated improvement and functionality. The Constant–Murley and American Shoulder and Elbow Society (ASES) shoulder scale indicated progress as scores improved. Pain scales involving both scores demonstrated improvement as well. One patient out of thirteen reported persistent pain despite improvement in the Constant–Murley score and successful MRIs. Four patients experienced complications such as shoulder inflammation during arthroscopy, bursitis, rupture of the bicep’s long head, and adhesive capsulitis. These complications were resolved along with satisfactory healing of the PTT with no evidence of the scaffold causing these [79]. The 5-year follow-up showed improvement in the Constant–Murley and ASES scales. Apart from the complications from the initial study, no further adverse effects were observed due to the scaffold. Degenerative rotator cuff tears progressed in two patients as identified by MRI. The collagen implant induced healing of the original defect but was unable to prevent progressive underlying disease [78,81].

Evaluating complications and outcomes in FTT repair by Bokor et al. [80] indicate an overall positive outcome. The rotator cuffs were intact with comprehensive tendon repair at the implantation sites as demonstrated by patient MRIs. Constant–Murley and ASES scores increased significantly over the 24 months along with a significant decrease on the pain scale post-operatively. However, one patient presented with pain at 26 months with no evidence of a faulty repair. Additionally, a patient had capsulitis pre-operatively which persisted for three months with improvement. The authors noted no adverse effects as a result of the collagen scaffold implantation [80].

Clinical assessment was similar in Thon et al. [80], however, ASES scores were not taken pre-and post-operatively. The overall ASES shoulder score across all groups was 82.87 with no difference between large and massive tears. One patient had a healing failure which was determined by MRI at 3 months in the supraspinatus tendon due to lack of repair. Another patient had progressive arthritis and atrophy of the rotator cuff muscles which required reverse shoulder arthroplasty 25 months post-operatively. However, it was noted by the authors that the patient’s rotator cuff tear healed but the scaffold had no impact on her osteoarthritis and atrophy. Eight patients had post-operative scapular dyskinesia, but this was not attributed to the scaffold. Overall, the collagen scaffold implanted by Thon et al. is indeed biocompatible and regenerative for shoulder augmentation [81].

In the study by Schlegel et al. [80], ASES pain and shoulder scores improved post-operatively by 1 year. Constant–Murley scores improved to 81.4 by 1 year post-operatively. The 1-year evaluation indicated that 94% of the patients were satisfied with the results of the augmentation. Regardless, complications such as subacromial fluid buildup, pain, dermatological reactions, and a case involving cardiac ablation were reported. The patients were treated accordingly, and the complaints were resolved with medication or surgically. These reactions were not linked to the presence of the collagen implant and no device-related adverse effects were reported [82].

Dai et al. [83] and Bushnell et al. [83] used the Smith and Nephew Regeneten scaffold for PTT and FTT repair, respectively. Dai et al. utilized ASES and Visual Analog Scale (VAS) scores for functional assessment pre- and post-operatively. ASES and VAS scores improved significantly at the 1-year follow up, indicating greater shoulder function and less pain. ASES improved from 45.6 to 68.1, and VAS improved from 8.3 to 3.8 post-operatively. Patients rated satisfaction at 7.5 out of 10 post-operatively. One complication emerged when a patient encountered a traumatic re-tear four months post-operatively unrelated to implantation [83]. Bushnell’s larger sample had 19 re-tears at the 1-year follow-up. Variance in surgical technique produced a greater number of re-tears with single-row repair having greater rates compared to double-row repair. ASES and Constant–Murley scores improved within one year as patients met the minimal clinically important difference. Likewise, 96.5% of patients were satisfied with the results of the surgery. Seven reoperations were performed for revision rotator cuff repair due to re-tearing but were not related to the implant. Two reoperations may have been linked to the collagen scaffold, procedure, or anchors as the first patient experienced swelling and a possible infection post-operatively and thus required debridement. The second patient experienced inflammation and osteopenia in the greater tuberosity leading to future debridement and aspiration [84]. Despite the complications, the overall result of the Regeneten collagen implant is positive and demonstrates good tendon healing in the cohort.

### 4.2. ECM-Based Scaffolds

Iannotti et al. [85] demonstrated no clinical benefit from porcine SIS scaffolds. Specifically, the median post-operative PENN shoulder score was greater in the control group with 91 points. Patient satisfaction was at 10 and shoulder functionality was at 59 with fully healed rotator cuffs. Shoulder functionality was decreased in the augmentation group, but the median PENN score was not significant between both groups. Adverse effects were present in three patients in the augmentation group. One patient experienced erythema with spontaneous drainage which required debridement with tissue loss present at the posterior edge of the infraspinatus. Another patient experienced erythema and greater temperature near the wound which resolved without intervention. The third patient experienced swelling and pain requiring aspiration of the subacromial space. All adverse events were resolved with tendon healing by the 1-year checkup [85].

The clinical implications reported by Bryant et al. [86] with porcine SIS scaffolds indicate a slight effect bias towards the control group. Patients reported a similar range of motion and strength in both groups along with progressing Western Ontario Rotator Cuff Index scores (WORC) over 24 months. Two patients in the experimental group required surgical intervention as one had a deep infection, and another experienced a biceps tendon rupture post-operatively. Warmth near the surgical site and fever were also present in two patients resolving without intervention. The control group with 3 patients experienced adverse events. One patient experienced an infection that was treated by antibiotics, and the others required surgical repair or shoulder manipulation. These results are concurrent with Iannotti et al. [86] as it is unlikely to have clinical benefit from SIS scaffolds [86].

Castagna et al. [87] utilized the Constant–Murley evaluation to determine shoulder functionality in both the control and experimental groups. Scores taken before surgery were lower than post-operative scores, but at the 24-month evaluation were significantly higher in the treatment group with the mean being 71.4 compared to 63.9 in the control. Patients with re-tearing in the experimental group had significant functionality compared to patients with re-tears in the control. No adverse events were noted and there was no indication of tissue rejection indicating that the porcine dermal-derived ECM scaffolds are safe and effective for improving shoulder functionality [87]. This, however, is limited to the small sample size which requires studies with larger patient population for determining the exact efficacy.

A study by Avanzi et al. [88] evaluating the effectiveness of a porcine dermal patch produced excellent clinical results. The authors employed the EuroQol-Visual Analog Scale (EQ-VAS), Disabilities of the Arm, Shoulder, and Hand (DASH), Simple Shoulder Test (SST), and Constant–Murley scores to determine the functionality of the augmentation. The EQ-VAS, DASH, and SST scores reflected no significant difference between groups but were significant in improvement from pre- to post-operative scores. Specifically, Constant–Murley scores improved post-operatively in both groups with scores higher in the augmentation group at 95.5 at 24 months, indicating that shoulder functionality improved with the porcine dermal patch. Four patients were classified as “not healed” and two patients experienced a re-tear. Furthermore, two patients experienced complications unrelated to the porcine dermal patch [88]. The clinical assessment of this clinical trial provides promising results, despite its contradictory results when compared to those of Maillot et al. [89]. The clinical outcomes indicate no difference between Constant–Murley scores in each group, but scores increased post-operatively. There was a significant difference between (1) patch and debridement and (2) repair and debridement groups at 12 and 24 months. Similarly, no difference was noted for VAS and active mobility between groups. Complications arose in the augmentation and arthroscopic repair group. One patient experienced a deep infection that required removal of the patch and four others experienced post-operative stiffness which improved within 12 months in the augmentation group. One patient in the arthroscopic repair group experienced a superficial wound infection that cleared after antibiotic treatment [89].

Shoulder assessment conducted by Gouk et al. [91] in the use of human acellular dermal patches were measured by DASH, Constant–Murley, and Oxford shoulder scores. Average scores were 48.2, 37.16, and 24.94 for Constant–Murley, Oxford, and DASH, respectively. The majority of patients were also satisfied with surgery, however, MRI results by 6 months indicated graft failure. Also, one patient experienced an infection that required the removal of the graft [90]. The patients evaluated by Johnson et al. [91] had greater Constant–Murley scores in the non-operated shoulder, but function in the augmented shoulder was satisfactory. There was no difference between Oxford and DASH scores between operated and non-operated shoulders. No complications were recorded from the scaffold [91]. The satisfactory results and intact grafts detected by ultrasound from Johnson et al. are in contrast with the graft failure and complications reported by Gouk et al. [91].

### 4.3. Synthetic-Based Scaffolds

FTT repair by Encalada-Diaz et al. [92] with the polycarbonate polyurethane scaffold showed promising clinical results with VAS (2.6), SST (7.7), and ASES (73.3) scores all significantly higher post-operatively at the 1-year follow-up. Also, the Constant Activities of Daily Living (CADL) and UCLA scores improved post-operatively from 6 to 12 months. No complications were found associated with the synthetic patch and no inflammatory response was present [92]. This is a significant and promising finding because no adverse reaction was present despite the presence of the synthetic patch, thus, indicating its clinical efficacy.

The use of the polyurethane urea scaffold by Petriccioli et al. [93] resulted in improved VAS, DASH, and Constant–Murley scores. Patients experienced improvements post-operatively in shoulder function by 23 months. Additionally, functional use significant improvement in the belly-press, lift-off, and bear-hug test indicating subscapular function was re-emerging. The re-tear rate was at 10% with no complications present in any patient. No infection or inflammation was present as a result of the exposure to the polyurethane urea scaffold [93], again indicating promising results in subscapularis repairs.

Another study utilized ASES scores to evaluate shoulder functionality post-operatively. There was a significant improvement in ASES scores 6 to 42 months after surgery. The final score was 82 at 42 months. Moreover, ASES scores were significantly higher for patients with an intact repair compared to a re-tear by 42 months. No adverse events or tissue rejections were reported due to exposure to the poly-L-lactic acid scaffold [66], indicating satisfactory clinical results in shoulder functionality.

The results of Ciampi et al. [94] indicate an excellent clinical outcome with the use of polypropylene scaffolds. The re-tear rate was low in addition to improvement in shoulder functionality. VAS scores improved overall over the 36 months. UCLA scores were significantly better in the propylene group at 36 months with the mean being 24.61 as compared to the control and collagen groups. No adverse events or inflammatory reactions were detected from the augmentation [94]. The overall results are more favorable toward the usage of polypropylene compared to the biological collagen for augmentation. Furthermore, the clinical assessment of Vitali et al. [94] supports the study of Ciampi et al. [94]. Similarly, this study also reported improvements in the VAS and UCLA over 36 months. The post-operative UCLA score for the propylene group was 24.6 compared to 14.73 in the control. Patient satisfaction was recorded in 52 patients with no foreign-body reactions or adverse events recorded [95].

Constant–Murley and Subjective Shoulder Value (SSV) scores improved significantly by the final 22-month follow-up in a study conducted by Smolen et al. [94] Reports of pain improved by the final follow-up. There was high patient satisfaction since shoulder functionality was also restored. In the cases of re-tearing, one involved detachment of the polyester while it remained attached in the other six cases. One patient experienced crepitus requiring the scaffold to be removed. There was no evidence of a foreign body reaction with no other adverse events attributed to the scaffold [96]. The clinical implications of the polyester patch indicate substantial functionality and lower re-tear rates. This is reiterated by another study where polyester scaffolds were tested in a smaller sample size (n = 29). Greater improvements were present in the group with the polyester as compared to the control by 6 months. Both the Oxford score and the Shoulder Pain and Disability Index (SPADI) improved significantly in the patch group. Constant–Murley scores were similar across groups while EQ-VAS was improved with the polyester patch. No adverse events were reported due to the polyester [97]. The concurrent results of these studies indicate favorability for polyester patches in both small and medium sample sizes for augmentation.

## 5. Discussion and Future Prospective

Gel-based composite scaffolds represent a promising avenue in tissue engineering, combining mechanical and biological innovative formulations and designs to address the complex requirements of rotator cuff repair. Ongoing research aims to optimize these scaffolds for clinical use, ensuring effective and successful tissue regeneration. Recent progress involves the development of composite scaffolds that combine hydrogels with reinforcing materials like ceramics or synthetic polymers [24,115]. This approach addresses the mechanical challenges of rotator cuff repair by providing a stiffness gradient that mimics the natural transition from tendon to bone [54,55]. Nanofibers and nanoparticles are also being integrated into gel-based scaffolds to enhance their mechanical strength and fatigue resistance while maintaining elasticity [56,116].

Advanced manufacturing technology like Electrospinning and 3D printing has further advanced the fabrication of scaffolds with custom designed and highly organized structures and controlled porosity, allowing for better nutrient diffusion, cell infiltration and angiogenesis [34,39]. These scaffolds often include biodegradable materials that degrade at controlled rates, ensuring temporary mechanical support as cells begin to produce native ECM and new tissue forms. Moreover, researchers are exploring drug delivery capabilities within these scaffolds to release anti-inflammatory or antimicrobial agents, reducing post-operative complications and improving healing outcomes [27].

However, these scaffolds also have limitations. Hydrogels alone lack sufficient mechanical strength and often require reinforcement with polymers or ceramics [24], which can complicate their design. Achieving an optimal degradation rate remains challenging since premature or delayed scaffold breakdown can hinder healing [39,117]. Also, complex manufacturing processes and high production costs limit scalability and accessibility. Furthermore, despite promising preclinical results, clinical data on large-scale efficacy and safety are still limited [118]. Some advanced components, like nanoparticles, may pose a risk of localized inflammation or immune responses. Addressing these challenges is essential in order to make these tissue engineered scaffolds viable for widespread clinical use.

Overall, gel-based scaffolds represent a promising approach to enhancing rotator cuff repair. Gel-based scaffolds with bioactive molecules such as genes, growth factors or small molecules can further enhance their healing potential. For example, incorporating tendon-specific growth factors like tendon-derived growth factor (TDGF) could improve the regeneration of tendon tissues [119]. Combining gel scaffolds with stem cells (e.g., mesenchymal stem cells or tendon-derived stem cells) holds significant potential for improving healing and regeneration [120]. The scaffold provides biomechanical support while the stem cells differentiate into tenocytes, accelerating tissue repair. In the future, greater advances in tissue engineering might lead to the development of personalized scaffolds tailored to an individual’s specific injury, improving the chances of successful rotator cuff repair.

## 6. Conclusions

Advances in orthopedic surgery have allowed individuals to regain shoulder functionality after rotator cuff tears. More recently, gel-based composite scaffolds offer significant promise in improving the clinical outcomes of rotator cuff repair. Their ability to promote tendon regeneration, support cell migration, prevent immune reaction, and reduce complications makes them a valuable adjunct to surgical repair, especially for large or difficult-to-heal tears. Although some biomaterials such as porcine SIS grafts may provoke an immune reaction, a wide array of others such as collagen-based, ECM-based and synthetic-based composites can be used as alternatives. The clinical implications of using such scaffolds/patches are beneficial as shoulder functionality, ROM, and pain improves following augmentation. Moreover, adverse events and re-tear rates appear to be low which further encourages the use of such hydrogel-based composite scaffolds. Furthermore, the incorporation of stem cells and bioactive molecules such as growth factors, hormones, and drugs into the scaffolds represents a significant technological advancement, enhancing scaffold performance in rotator cuff repair and other tissue engineering applications. The future of shoulder rotator cuff repair looks very promising with the use of these various types of tissue-engineered composite scaffolds. But, searching clinicaltrials.gov with the “term rotator cuff tears” and “scaffold” indicates only two active and two recruiting clinical trials using tissue-engineered scaffolds. Ten other studies that were also listed are either completed or withdrawn. Clearly, given this small number of active clinical trials, many more are warranted. Regardless, this review serves as a quick guide for clinical evaluation research of novel surgical or regenerative material options for rotator cuff treatment.

## Figures and Tables

**Table 1:** Materials of comparative characteristics for gel-based composite scaffolds.

Material	Mechanical Strength	Biological Activity	Degradation Rate	Clinical Application
**Collagen-based [25]**	Low (enhanced with crosslinking)	High (cell adhesion and migration)	Moderate (enzymatic)	Ideal for tendon regeneration
**Hyaluronic Acid [26,27]**	Low (soft and hydrophilic)	High (anti-inflammatory)	Fast (enzymatic)	Supports healing and reduces inflammation
**Alginate-based [28]**	Moderate (crosslinking enhances)	Low (requires modification)	Moderate (enzyme-controlled)	Good for cell encapsulation
**Fibrin-based [29]**	Moderate (initial strength)	High (promotes healing and angiogenesis)	Moderate (fibrinolysis)	Wound healing and tendon-to-bone integration
**PEG-based [30]**	High (tunable)	Low (requires functionalization)	Slow (stable, controlled)	Ideal for mechanical scaffold designs
**PLGA-based [23,31]**	High (strong and load bearing)	Low (requires biofunctionalization)	Moderate (hydrolytic)	Load-bearing, controlled degradation
**Composite (e.g., Collagen + PEG) [25,32]**	High (balanced strength)	High (synergistic effect)	Controlled (depends on components)	Combines strength and biological support

**Table 2:** Manufacturing processes used for the production of gel-based composite scaffolds.

Manufacturing Process	Advantages	Challenges	Ideal Applications
**Freeze-Drying [33]**	High porosity, retains material structure, customizable pore size	Mechanical weakness, complex process	Soft tissue scaffolds, natural polymer scaffolds (collagen, alginate)
**Electrospinning [34,35]**	High surface area, fiber alignment, ECM mimicry	Mechanical weakness, scalability issues	Tendon, ligament tissue engineering, rotator cuff repairs
**3D Bioprinting [36,37,38,39]** **(Biofabrication)**	High precision, patient-specific, complex geometry	High cost, slow production speed, bioink challenges	Patient-specific scaffolds for rotator cuff repair, bone, cartilage
**Solvent Casting and Particulate Leaching [40,41]**	Simple, cost-effective, porosity control	Mechanical strength, solvent residue	Soft tissue regeneration, muscle and tendon scaffolds
**Self-Assembly and Crosslinking [42,43]**	Mimics natural processes, high biocompatibility	Lack of reproducibility, weak strength	Collagen-based scaffolds, tendon and ligament repair
**Gelation [44]**	Minimally invasive, customizable shape, ease of use	Weak mechanical properties, gelation consistency issues	Injectable scaffolds for rotator cuff and soft tissue repair
**Smart Scaffolds [45]**	Controlled release of growth factors, tailored response	Complexity in design and fabrication	Rotator cuff and tendon regeneration
**Hybrid Scaffolds** **(Natural + Synthetic) [46,47]**	Combines the best of both materials (biocompatibility + strength)	Complexity in material blending and manufacturing	Used for load-bearing rotator cuff repair
**Injectable Hydrogels [48,49]**	Minimally invasive delivery and customization	Mechanical strength may not be sufficient	Ideal for soft tissue repair and rotator cuff

**Table 3:** Mechanical properties of gel-based composite scaffold.

Property	Description	Purpose/Benefit
**Elasticity**	Matches the flexibility of native tendons. Elastic moduli for human tendons range from 1.2 to 1.8 GPa [57].	Reduces stress concentrations and supports biomechanical compatibility.
**Tensile Strength**	Enhanced through reinforcement with polymers or ceramics. Ranges from 45 to 150 MPa for human tendons [57,58].	Supports the mechanical loads during tendon movement.
**Stiffness Gradient**	Gradual transition from soft (gel-based) to stiff (ceramic/polymer-based) zones.	Mimics the natural tendon-to-bone interface, ensuring smooth load transfer.
**Load-Bearing Capability**	Improved with composite materials like hydroxyapatite or nanofibers.	Withstands physiological loads, ensuring durability during the healing process.
**Compression Resistance**	Enhanced resistance to compressive forces. Compressive strength of cortical bone is typically 100–200 MPa [59].	Useful for distributing loads at the tendon-bone junction.
**Porosity**	Desired mechanical properties with controlled porosity, and optimal pore size.	Porosity impacts the material’s strength and facilitate better cell and tissue integration.
**Degradation Rate**	Designed to degrade gradually, allowing for tissue formation and integration.	Degradation process can be influenced by the gel matrix (e.g., natural versus synthetic polymers) and the crosslinking agents used to stabilize the scaffold.
**Fatigue Resistance**	Improved by adding nanoscale fillers (e.g., graphene oxide).	Ensures performance under repetitive mechanical stresses over extended periods.
**Durability**	Maintains structural integrity over time with reinforced hydrogels.	Reduces risk of scaffold failure during tissue regeneration.

**Table 4:** Summary of studies.

Study	Material	Summary of Findings
**Collagen-based Augmentation**		
Bokor [78,79,80]	Collagen	↑ Tendon thickness; reduced defect size
	Collagen *	No degeneration observed; tendon thickness better than pre-operative
	Collagen	↑ Tendon thickness and integration
Thon [81]	Collagen	↑ Tendon thickness and vascularity; 96% of patients healed
Schlegel [82]	Collagen	↓ Defect size for 70% of patients; ↑ Tendon thickness
Dai [83]	Collagen **	↑ Tendon thickness
Bushnell [84]	Collagen	Integration present and lower re-tear rates
**ECM-based Augmentation**		
Iannotti [85]	SIS **	Less healing in massive tears; no clear clinical benefit
Bryant [86]	SIS **	52.9% augmentation failure; no clear clinical benefit
Castagna [87]	DDCM **	Fewer re-tears, determined to be safe
Avanzi [88]	Dermal Patch **	97.6% healing with patch; ↑ Tendon thickness and strength recovery
Maillot [89]	Dermal Patch **	No superior benefit of use; slower anterior elevation, and external rotation recovery
Gouk [90]	Acellular Dermal Patch ***	86% of grafts failed; failed biocompatibility
Johnson [91]	Dermal Patch ***	Intact grafts by 35 months; functional ROM
**Synthetic-based Augmentation**		
Encalada-Diaz [92]	Polycarbonate Polyurethane	90% of tears healed; ROM recovered
Petriccioli [93]	Polyurethane	Greater tendon thickness in repaired shoulder compared to healthy shoulder
Proctor [66]	PLLA	83% repair rate (12 months); 78% (48 months), ↑ Tendon integrity
Ciampi [94]	Propylene	Fewer re-tears than the collagen group; stronger abduction strength and elevation
Vitali [95]	Propylene	Greater abduction and scapular plane elevation, low retears; ↑ Tendon integrity
Smolen [96]	Polyester	86% of repairs intact, 14% re-tear; significant improvement in internal rotation
Cowling [97]	Polyester	Improved shoulder functionality; no difference in Goutallier classification

* 5-year follow-up study on Bokor et al. [78]; ** porcine xenograft; *** human allograft; DDCM, dermal-derived collagen membrane; SIS, small intestine submucosa; PLLA, poly-L-lactic acid.

**Table 5:** Clinical implications in treatment groups.

Study	Material	Patients Treated (n)	AE (n)	Functionality	Level
**Collagen-based Augmentation**					
Bokor [78,79,80]	Collagen	13	4	↑ CM	-
	Collagen *	11	0	↑ CM, ASES	IV
	Collagen	9	2	↑ CM, ASES, ↓ Pain	-
Thon [81]	Collagen	23	9	High ASES scores (82.87)	IV
Schlegel [82]	Collagen	33	5	↑ CM, ASES, ↓ Pain	IV
Dai [83]	Collagen **	30	1	↑ ASES, VAS	-
Bushnell [84]	Collagen	115	7, 2 †	↑ CM, ASES	IV
**ECM-based Augmentation**					
Iannotti [85]	SIS **	15	3	↓ PENN, No clinical benefit	II
Bryant [86]	SIS **	34	4	52.9% risk of failure, No clinical benefit	I
Castagna [87]	DDCM **	35	0	↑ CM, More functionality in treatment	III
Avanzi [88]	Dermal Patch **	46	2	↑ EQ-VAS, DASH, SST, CM post-operative	II
Maillot [89]	Dermal Patch **	11	4, 1 †	↑ CM post-operative, No difference in VAS/mobility	II
Gouk [90]	Acellular Dermal Patch ***	8	1	No difference in Oxford/DASH, No clinical benefit	-
Johnson [91]	Dermal Patch ***	14	0	No difference in Oxford/DASH	IV
**Synthetic-based Augmentation**					
Encalada-Diaz [92]	Polycarbonate Polyurethane	10	0	↑ VAS, SST, ASES, CADL, UCLA	IV
Petriccioli [93]	Polyurethane	10	0	↑ VAS, DASH, CM, subscapular function	IV
Proctor [66]	PLLA	18	0	↑ ASES	IV
Ciampi [94]	Propylene	52	0	↑ VAS, UCLA	III
Vitali [95]	Propylene	60	0	↑ VAS, UCLA	-
Smolen [96]	Polyester	50	0	↑ CM, SSV, ↓ Pain,	IV
Cowling [97]	Polyester	29	0	↑ Oxford, SPADI, EQ-VAS, CM similar across groups	-

* 5-year follow-up study on Bokor et al.; ** porcine xenograft; *** human allograft; † complications may be attributed to the implant, otherwise unlinked; AE, adverse events; DDCM, dermal-derived collagen membrane; SIS, small intestine submucosa; PLLA, poly-L-lactic acid; CM, Constant–Murley Score; ASES, American Shoulder and Elbow Society Shoulder Scale; VAS, visual analog scale; PENN, PENN Shoulder Score; EQ-VAS, EuroQol-Visual Analog Scale; DASH, disabilities of the arm, shoulder, and hand; SST, simple shoulder test; Oxford, Oxford Shoulder Score; CADL, constant activities of daily living; UCLA, UCLA Shoulder Score; SSV, subjective shoulder value; SPADI, shoulder pain and disability index.

## Data Availability

No new data were created or analyzed in this study. Data sharing is not applicable to this article.

## References

[1] May T., Garmel G.M. (2019). Rotator Cuff Injury. StatPearls [Internet].

[2] Papatheodorou A., Ellinas P., Takis F., Tsanis A., Maris I., Batakis N. (2006). US of the shoulder: Rotator cuff and non–rotator cuff disorders. Radiographics.

[3] Lugo R., Kung P., Ma C.B. (2008). Shoulder biomechanics. Eur. J. Radiol..

[4] Dabija D.I., Gao C., Edwards T.L., Kuhn J.E., Jain N.B. (2017). Genetic and familial predisposition to rotator cuff disease: A systematic review. J. Shoulder Elb. Surg..

[5] Minagawa H., Yamamoto N., Abe H., Fukuda M., Seki N., Kikuchi K., Kijima H., Itoi E. (2013). Prevalence of symptomatic and asymptomatic rotator cuff tears in the general population: From mass-screening in one village. J. Orthop..

[6] Tashjian R.Z., Farnham J.M., Albright F.S., Teerlink C.C., Cannon-Albright L.A. (2009). Evidence for an inherited predisposition contributing to the risk for rotator cuff disease. J. Bone Jt. Surg. Am. Vol..

[7] Gill T.K., Shanahan E.M., Tucker G.R., Buchbinder R., Hill C.L. (2020). Shoulder range of movement in the general population: Age and gender stratified normative data using a community-based cohort. BMC Musculoskelet. Disord..

[8] Gillooly J.J., Chidambaram R., Mok D. (2010). The lateral Jobe test: A more reliable method of diagnosing rotator cuff tears. Int. J. Shoulder Surg..

[9] Hughes P.C., Taylor N.F., Green R.A. (2008). Most clinical tests cannot accurately diagnose rotator cuff pathology: A systematic review. Aust. J. Physiother..

[10] Sawalha S., Fischer J. (2015). The accuracy of “subacromial grind test” in diagnosis of supraspinatus rotator cuff tears. Int. J. Shoulder Surg..

[11] Sambandam S.N., Khanna V., Gul A., Mounasamy V. (2015). Rotator cuff tears: An evidence based approach. World J. Orthop..

[12] Blevins F.T. (1997). Rotator cuff pathology in athletes. Sports Med..

[13] Teunis T., Lubberts B., Reilly B.T., Ring D. (2014). A systematic review and pooled analysis of the prevalence of rotator cuff disease with increasing age. J. Shoulder Elb. Surg..

[14] Kim H.M., Teefey S.A., Zelig A., Galatz L.M., Keener J.D., Yamaguchi K. (2009). Shoulder strength in asymptomatic individuals with intact compared with torn rotator cuffs. J. Bone Jt. Surg. Am. Vol..

[15] Yamaguchi K., Ditsios K., Middleton W.D., Hildebolt C.F., Galatz L.M., Teefey S.A. (2006). The demographic and morphological features of rotator cuff disease: A comparison of asymptomatic and symptomatic shoulders. J. Bone Jt. Surg. Am..

[16] Fukuda H. (2003). The management of partial-thickness tears of the rotator cuff. J. Bone Jt. Surg. Br. Vol..

[17] Clement N.D., Nie Y.X., McBirnie J.M. (2012). Management of degenerative rotator cuff tears: A review and treatment strategy. Sports Med. Arthrosc. Rehabil. Ther. Technol..

[18] Liem D., Alci S., Dedy N., Steinbeck J., Marquardt B., Möllenhoff G. (2008). Clinical and structural results of partial supraspinatus tears treated by subacromial decompression without repair. Knee Surg. Sports Traumatol. Arthrosc..

[19] Franceschi F., Papalia R., Palumbo A., Del Buono A., Maffulli N., Denaro V. (2012). Operative management of partial-and full-thickness rotator cuff tears. Rotator Cuff Tear.

[20] Cofield R.H., Parvizi J., Hoffmeyer P.J., Lanzer W.L., Ilstrup D.M., Rowland C.M. (2001). Surgical repair of chronic rotator cuff tears: A prospective long-term study. J. Bone Jt. Surg. Am..

[21] Patel S., Gualtieri A.P., Lu H.H., Levine W.N. (2016). Advances in biologic augmentation for rotator cuff repair. Ann. N. Y. Acad. Sci..

[22] Maisani M., Ziane S., Ehret C., Levesque L., Siadous R., Le Meins J.F., Chevallier P., Barthélémy P., De Oliveira H., Amédée J. (2018). A new composite hydrogel combining the biological properties of collagen with the mechanical properties of a supramolecular scaffold for bone tissue engineering. J. Tissue Eng. Regen. Med..

[23] Rahman C.V., Kuhn G., White L.J., Kirby G.T., Varghese O.P., McLaren J.S., Cox H.C., Rose F.R., Müller R., Hilborn J. (2013). PLGA/PEG-hydrogel composite scaffolds with controllable mechanical properties. J. Biomed. Mater. Res. Part B Appl. Biomater..

[24] Suvarnapathaki S., Wu X., Lantigua D., Nguyen M.A., Camci-Unal G. (2020). Hydroxyapatite-incorporated composite gels improve mechanical properties and bioactivity of bone scaffolds. Macromol. Biosci..

[25] Zhang D., Wu X., Chen J., Lin K. (2018). The development of collagen based composite scaffolds for bone regeneration. Bioact. Mater..

[26] Collins M.N., Birkinshaw C. (2013). Hyaluronic acid based scaffolds for tissue engineering—A review. Carbohydr. Polym..

[27] Wang S., Lei H., Mi Y., Ma P., Fan D. (2024). Chitosan and hyaluronic acid based injectable dual network hydrogels-Mediating antimicrobial and inflammatory modulation to promote healing of infected bone defects. Int. J. Biol. Macromol..

[28] Venkatesan J., Bhatnagar I., Manivasagan P., Kang K.-H., Kim S.-K. (2015). Alginate composites for bone tissue engineering: A review. Int. J. Biol. Macromol..

[29] Noori A., Ashrafi S.J., Vaez-Ghaemi R., Hatamian-Zaremi A., Webster T.J. (2017). A review of fibrin and fibrin composites for bone tissue engineering. Int. J. Nanomed..

[30] Kutikov A.B., Song J. (2015). Biodegradable PEG-based amphiphilic block copolymers for tissue engineering applications. ACS Biomater. Sci. Eng..

[31] Chen P., Cui L., Chen G., You T., Li W., Zuo J., Wang C., Zhang W., Jiang C. (2019). The application of BMP-12-overexpressing mesenchymal stem cells loaded 3D-printed PLGA scaffolds in rabbit rotator cuff repair. Int. J. Biol. Macromol..

[32] Pazarçeviren E., Erdemli Ö., Keskin D., Tezcaner A. (2017). Clinoptilolite/PCL–PEG–PCL composite scaffolds for bone tissue engineering applications. J. Biomater. Appl..

[33] Teimouri A., Azadi M., Emadi R., Lari J., Chermahini A.N. (2015). Preparation, characterization, degradation and biocompatibility of different silk fibroin based composite scaffolds prepared by freeze-drying method for tissue engineering application. Polym. Degrad. Stab..

[34] Lim T.K., Dorthé E., Williams A., D D’Lima D. (2021). Nanofiber scaffolds by electrospinning for rotator cuff tissue engineering. Chonnam Med. J..

[35] Orr S.B., Chainani A., Hippensteel K.J., Kishan A., Gilchrist C., Garrigues N.W., Ruch D.S., Guilak F., Little D. (2015). Aligned multilayered electrospun scaffolds for rotator cuff tendon tissue engineering. Acta Biomater..

[36] Singh S., Ramakrishna S., Berto F. (2020). 3D Printing of polymer composites: A short review. Mater. Des. Process. Commun..

[37] Chen X., Anvari-Yazdi A.F., Duan X., Zimmerling A., Gharraei R., Sharma N., Sweilem S., Ning L. (2023). Biomaterials/bioinks and extrusion bioprinting. Bioact. Mater..

[38] Jiang X., Wu S., Kuss M., Kong Y., Shi W., Streubel P.N., Li T., Duan B. (2020). 3D printing of multilayered scaffolds for rotator cuff tendon regeneration. Bioact. Mater..

[39] Pahlevanzadeh F., Emadi R., Valiani A., Kharaziha M., Poursamar S.A., Bakhsheshi-Rad H.R., Ismail A.F., RamaKrishna S., Berto F. (2020). Three-dimensional printing constructs based on the chitosan for tissue regeneration: State of the art, developing directions and prospect trends. Materials.

[40] Deng Y., Zhang M., Chen X., Pu X., Liao X., Huang Z., Yin G. (2017). A novel akermanite/poly (lactic-co-glycolic acid) porous composite scaffold fabricated via a solvent casting-particulate leaching method improved by solvent self-proliferating process. Regen. Biomater..

[41] Thadavirul N., Pavasant P., Supaphol P. (2014). Development of polycaprolactone porous scaffolds by combining solvent casting, particulate leaching, and polymer leaching techniques for bone tissue engineering. J. Biomed. Mater. Res. Part A.

[42] Yu P., Bao R.-Y., Shi X.-J., Yang W., Yang M.-B. (2017). Self-assembled high-strength hydroxyapatite/graphene oxide/chitosan composite hydrogel for bone tissue engineering. Carbohydr. Polym..

[43] Turnbull G., Clarke J., Picard F., Riches P., Jia L., Han F., Li B., Shu W. (2018). 3D bioactive composite scaffolds for bone tissue engineering. Bioact. Mater..

[44] Liu X., Smith L.A., Hu J., Ma P.X. (2009). Biomimetic nanofibrous gelatin/apatite composite scaffolds for bone tissue engineering. Biomaterials.

[45] Yuan X., Zhu W., Yang Z., He N., Chen F., Han X., Zhou K. (2024). Recent advances in 3D printing of smart scaffolds for bone tissue engineering and regeneration. Adv. Mater..

[46] Zou L., Zhang Y., Liu X., Chen J., Zhang Q. (2019). Biomimetic mineralization on natural and synthetic polymers to prepare hybrid scaffolds for bone tissue engineering. Colloids Surf. B Biointerfaces.

[47] Funakoshi T., Majima T., Iwasaki N., Suenaga N., Sawaguchi N., Shimode K., Minami A., Harada K., Nishimura S.-I. (2005). Application of tissue engineering techniques for rotator cuff regeneration using a chitosan-based hyaluronan hybrid fiber scaffold. Am. J. Sports Med..

[48] Han B., Jones I.A., Yang Z., Fang W., Vangsness C.T. (2020). Repair of rotator cuff tendon defects in aged rats using a growth factor injectable gel scaffold. Arthrosc. J. Arthrosc. Relat. Surg..

[49] Chen C.-H., Chang C.-H., Wang K.-C., Su C.-I., Liu H.-T., Yu C.-M., Wong C.-B., Wang I.-C., Whu S.W., Liu H.-W. (2011). Enhancement of rotator cuff tendon–bone healing with injectable periosteum progenitor cells-BMP-2 hydrogel in vivo. Knee Surg. Sports Traumatol. Arthrosc..

[50] Narayanan G., Nair L.S., Laurencin C.T. (2018). Regenerative engineering of the rotator cuff of the shoulder. ACS Biomater. Sci. Eng..

[51] Tsekes D., Konstantopoulos G., Khan W.S., Rossouw D., Elvey M., Singh J. (2019). Use of stem cells and growth factors in rotator cuff tendon repair. Eur. J. Orthop. Surg. Traumatol..

[52] Stace E.T., Nagra N.S., Tiberwel S., Khan W., Carr A.J. (2018). The use of electrospun scaffolds in musculoskeletal tissue engineering: A focus on tendon and the rotator cuff. Curr. Stem Cell Res. Ther..

[53] Moffat K.L., Levine W.N., Lu H.H. In vitro evaluation of rotator cuff tendon fibroblasts on aligned composite scaffold of polymer nanofibers and hydroxyapatite nanoparticles. Proceedings of the Transactions of the 54th Orthopaedic Research Society.

[54] Chen Y., Li Y., Zhu W., Liu Q. (2024). Biomimetic gradient scaffolds for the tissue engineering and regeneration of rotator cuff enthesis. Biofabrication.

[55] Shang P., Xiang Y., Du J., Chen S., Cheng B., Li J., Yuan F. (2023). Gradient Bipolar Nanofiber Scaffolds with a Structure of Biomimetic Tendon-Bone Interface as Rotator Cuff Patches. ACS Appl. Polym. Mater..

[56] Liu J., Lin S., Liu X., Qin Z., Yang Y., Zang J., Zhao X. (2020). Fatigue-resistant adhesion of hydrogels. Nat. Commun..

[57] Kaya D.Ö. (2020). Architecture of tendon and ligament and their adaptation to pathological conditions. Comparative Kinesiology of the Human Body.

[58] Martin R.B., Burr D.B., Sharkey N.A., Fyhrie D.P., Martin R.B., Burr D.B., Sharkey N.A., Fyhrie D.P. (2015). Mechanical properties of ligament and tendon. Skeletal Tissue Mechanics.

[59] Ryan E., Yin S. (2022). Compressive strength of β-TCP scaffolds fabricated via lithography-based manufacturing for bone tissue engineering. Ceram. Int..

[60] Smith R., Carr A., Dakin S., Snelling S., Yapp C., Hakimi O. (2016). The response of tenocytes to commercial scaffolds used for rotator cuff repair. eCells Mater. J..

[61] Yokoya S., Mochizuki Y., Natsu K., Omae H., Nagata Y., Ochi M. (2012). Rotator cuff regeneration using a bioabsorbable material with bone marrow–derived mesenchymal stem cells in a rabbit model. Am. J. Sports Med..

[62] Zhao S., Zhao J., Dong S., Huangfu X., Li B., Yang H., Zhao J., Cui W. (2014). Biological augmentation of rotator cuff repair using bFGF-loaded electrospun poly (lactide-co-glycolide) fibrous membranes. Int. J. Nanomed..

[63] Longo U.G., Lamberti A., Maffulli N., Denaro V. (2010). Tendon augmentation grafts: A systematic review. Br. Med. Bull..

[64] Adams J.E., Zobitz M.E., Reach J.S., An K.-N., Steinmann S.P. (2006). Rotator cuff repair using an acellular dermal matrix graft: An in vivo study in a canine model. Arthrosc. J. Arthrosc. Relat. Surg..

[65] Schlegel T.F., Hawkins R.J., Lewis C.W., Motta T., Turner A.S. (2006). The effects of augmentation with Swine small intestine submucosa on tendon healing under tension: Histologic and mechanical evaluations in sheep. Am. J. Sports Med..

[66] Proctor C.S. (2014). Long-term successful arthroscopic repair of large and massive rotator cuff tears with a functional and degradable reinforcement device. J. Shoulder Elb. Surg..

[67] Washburn III R., Anderson T.M., Tokish J.M. (2017). Arthroscopic rotator cuff augmentation: Surgical technique using bovine collagen bioinductive implant. Arthrosc. Tech..

[68] Cheung E.V., Silverio L., Sperling J.W. (2010). Strategies in biologic augmentation of rotator cuff repair: A review. Clin. Orthop. Relat. Res..

[69] Montgomery S.R., Petrigliano F.A., Gamradt S.C. (2011). Biologic augmentation of rotator cuff repair. Curr. Rev. Musculoskelet. Med..

[70] Voss A., McCarthy M.B., Allen D., Cote M.P., Beitzel K., Imhoff A.B., Mazzocca A.D. (2016). Fibrin scaffold as a carrier for mesenchymal stem cells and growth factors in shoulder rotator cuff repair. Arthrosc. Tech..

[71] Street M., Thambyah A., Dray M., Amirapu S., Tuari D., Callon K.E., McIntosh J.D., Burkert K., Dunbar P.R., Coleman B. (2015). Augmentation with an ovine forestomach matrix scaffold improves histological outcomes of rotator cuff repair in a rat model. J. Orthop. Surg. Res..

[72] Derwin K.A., Codsi M.J., Milks R.A., Baker A.R., McCarron J.A., Iannotti J.P. (2009). Rotator cuff repair augmentation in a canine model with use of a woven poly-L-lactide device. J. Bone Jt. Surg. Am. Vol..

[73] Easley J., Puttlitz C., Hackett E., Broomfield C., Nakamura L., Hawes M., Getz C., Frankle M., Pierre P.S., Tashjian R. (2020). A prospective study comparing tendon-to-bone interface healing using an interposition bioresorbable scaffold with a vented anchor for primary rotator cuff repair in sheep. J. Shoulder Elb. Surg..

[74] Van Kampen C., Arnoczky S., Parks P., Hackett E., Ruehlman D., Turner A., Schlegel T. (2013). Tissue-engineered augmentation of a rotator cuff tendon using a reconstituted collagen scaffold: A histological evaluation in sheep. Muscles Ligaments Tendons J..

[75] Fernandez de Grado G., Keller L., Idoux-Gillet Y., Wagner Q., Musset A.-M., Benkirane-Jessel N., Bornert F., Offner D. (2018). Bone substitutes: A review of their characteristics, clinical use, and perspectives for large bone defects management. J. Tissue Eng..

[76] Baldwin M., Nagra N., Greenall G., Carr A.J., Beard D., Rees J., Rangan A., Merritt N., Dritsaki M., Hopewell S. (2020). Use of implantable meshes for augmented rotator cuff repair: A systematic review and meta-analysis. BMJ Open.

[77] Thangarajah T., Pendegrass C.J., Shahbazi S., Lambert S., Alexander S., Blunn G.W. (2015). Augmentation of rotator cuff repair with soft tissue scaffolds. Orthop. J. Sports Med..

[78] Bokor D., Sonnabend D., Deady L., Cass B., Young A., Van Kampen C., Arnoczky S. (2019). Healing of partial-thickness rotator cuff tears following arthroscopic augmentation with a highly-porous collagen implant: A 5-year clinical and MRI follow-up. Muscles Ligaments Tendons J. MLTJ.

[79] Bokor D.J., Sonnabend D., Deady L., Cass B., Young A., Van Kampen C., Arnoczky S. (2016). Evidence of healing of partial-thickness rotator cuff tears following arthroscopic augmentation with a collagen implant: A 2-year MRI follow-up. Muscles Ligaments Tendons J..

[80] Bokor D.J., Sonnabend D., Deady L., Cass B., Young A., Van Kampen C., Arnoczky S. (2015). Preliminary investigation of a biological augmentation of rotator cuff repairs using a collagen implant: A 2-year MRI follow-up. Muscles Ligaments Tendons J..

[81] Thon S.G., O’Malley L., O’Brien M.J., Savoie III F.H. (2019). Evaluation of healing rates and safety with a bioinductive collagen patch for large and massive rotator cuff tears: 2-year safety and clinical outcomes. Am. J. Sports Med..

[82] Schlegel T.F., Abrams J.S., Bushnell B.D., Brock J.L., Ho C.P. (2018). Radiologic and clinical evaluation of a bioabsorbable collagen implant to treat partial-thickness tears: A prospective multicenter study. J. Shoulder Elb. Surg..

[83] Dai A.Z., Campbell A.L., Bloom D.A., Baron S.L., Begly J.P., Meislin R.J. (2020). Collagen-Based Bioinductive Implant for Treatment of Partial Thickness Rotator Cuff Tears. Bull. NYU Hosp. Jt. Dis..

[84] Bushnell B.D., Connor P.M., Harris H.W., Ho C.P., Trenhaile S.W., Abrams J.S. (2021). Re-tear rates and clinical outcomes at 1 year following repair of full-thickness rotator cuff tears augmented with a bioinductive collagen implant: A prospective multicenter study. JSES Int..

[85] Iannotti J.P., Codsi M.J., Kwon Y.W., Derwin K., Ciccone J., Brems J.J. (2006). Porcine small intestine submucosa augmentation of surgical repair of chronic two-tendon rotator cuff tears: A randomized, controlled trial. J. Bone Jt. Surg. Am..

[86] Bryant D., Holtby R., Willits K., Litchfield R., Drosdowech D., Spouge A., White D., Guyatt G. (2016). A randomized clinical trial to compare the effectiveness of rotator cuff repair with or without augmentation using porcine small intestine submucosa for patients with moderate to large rotator cuff tears: A pilot study. J. Shoulder Elb. Surg..

[87] Castagna A., Cesari E., Di Matteo B., Osimani M., Garofalo R., Kon E., Marcacci M., Chillemi C. (2018). Porcine dermal xenograft as augmentation in the treatment of large rotator cuff tears: Clinical and magnetic resonance results at 2-year follow-up. Joints.

[88] Avanzi P., Dei Giudici L., Capone A., Cardoni G., Lunardi G., Foti G., Zorzi C. (2019). Prospective randomized controlled trial for patch augmentation in rotator cuff repair: 24-month outcomes. J. Shoulder Elb. Surg..

[89] Maillot C., Harly E., Demezon H., Le Huec J.-C. (2018). Surgical repair of large-to-massive rotator cuff tears seems to be a better option than patch augmentation or debridement and biceps tenotomy: A prospective comparative study. J. Shoulder Elb. Surg..

[90] Gouk C.J.C., Shulman R.M., Buchan C., Thomas M.J.E., Taylor F.J. (2019). Failure of dermal allograft repair of massive rotator cuff tears in magnetic resonance imaging and clinical assessment. Clin. Orthop. Surg..

[91] Johnson S.M., Cherry J.V., Thomas N., Jafri M., Jariwala A., McLeod G.G. (2020). Clinical outcomes and ultrasonographic viability of GraftJacket^®^ augmented rotator cuff repair: A prospective follow-up study with mean follow-up of forty-one months. J. Clin. Orthop. Trauma.

[92] Encalada-Diaz I., Cole B.J., MacGillivray J.D., Ruiz-Suarez M., Kercher J.S., Friel N.A., Valero-Gonzalez F. (2011). Rotator cuff repair augmentation using a novel polycarbonate polyurethane patch: Preliminary results at 12 months’ follow-up. J. Shoulder Elb. Surg..

[93] Petriccioli D., Bertone C., Marchi G., Mujahed I. (2013). Open repair of isolated traumatic subscapularis tendon tears with a synthetic soft tissue reinforcement. Musculoskelet. Surg..

[94] Ciampi P., Scotti C., Nonis A., Vitali M., Di Serio C., Peretti G.M., Fraschini G. (2014). The benefit of synthetic versus biological patch augmentation in the repair of posterosuperior massive rotator cuff tears: A 3-year follow-up study. Am. J. Sports Med..

[95] Vitali M., Cusumano A., Pedretti A., Rodriguez N.N., Fraschini G. (2015). Employment of synthetic patch with augmentation of the long head of the biceps tendon in irreparable lesions of the rotator cuff: Our technique applied to 60 patients. Tech. Hand Up. Extrem. Surg..

[96] Smolen D., Haffner N., Mittermayr R., Hess F., Sternberg C., Leuzinger J. (2020). Application of a new polyester patch in arthroscopic massive rotator cuff repair—A prospective cohort study. J. Shoulder Elb. Surg..

[97] Cowling P., Hackney R., Dube B., Grainger A., Biglands J., Stanley M., Song D., Conaghan P., Kingsbury S. (2020). The use of a synthetic shoulder patch for large and massive rotator cuff tears–a feasibility study. BMC Musculoskelet. Disord..

[98] Smith M.J., Bozynski C.C., Kuroki K., Cook C.R., Stoker A.M., Cook J.L. (2020). Comparison of biologic scaffolds for augmentation of partial rotator cuff tears in a canine model. J. Shoulder Elb. Surg..

[99] Bishop J., Klepps S., Lo I.K., Bird J., Gladstone J.N., Flatow E.L. (2006). Cuff integrity after arthroscopic versus open rotator cuff repair: A prospective study. J. Shoulder Elb. Surg..

[100] Jo C.H., Park J.W., Shin J.S. (2016). Changes of muscle atrophy according to the immediate postoperative time point in magnetic resonance imaging after arthroscopic rotator cuff repair. Arthrosc. J. Arthrosc. Relat. Surg..

[101] Rashid M.S., Cooper C., Cook J., Cooper D., Dakin S.G., Snelling S., Carr A.J. (2017). Increasing age and tear size reduce rotator cuff repair healing rate at 1 year: Data from a large randomized controlled trial. Acta Orthop..

[102] Sugaya H., Maeda K., Matsuki K., Moriishi J. (2007). Repair integrity and functional outcome after arthroscopic double-row rotator cuff repair: A prospective outcome study. J. Bone Jt. Surg..

[103] Taniguchi N., Suenaga N., Oizumi N., Miyoshi N., Yamaguchi H., Inoue K., Chosa E. (2015). Bone marrow stimulation at the footprint of arthroscopic surface-holding repair advances cuff repair integrity. J. Shoulder Elb. Surg..

[104] Derwin K.A., Badylak S.F., Steinmann S.P., Iannotti J.P. (2010). Extracellular matrix scaffold devices for rotator cuff repair. J. Shoulder Elb. Surg..

[105] Goldenberg B.T., Lacheta L., Dekker T.J., Spratt J.D., Nolte P.C., Millett P.J. (2020). Biologics to Improve Healing in Large and Massive Rotator Cuff Tears: A Critical Review. Orthop. Res. Rev..

[106] Malcarney H.L., Bonar F., Murrell G.A. (2005). Early inflammatory reaction after rotator cuff repair with a porcine small intestine submucosal implant: A report of 4 cases. Am. J. Sports Med..

[107] Phipatanakul W.P., Petersen S.A. (2009). Porcine small intestine submucosa xenograft augmentation in repair of massive rotator cuff tears. Am. J. Orthop..

[108] Walton J.R., Bowman N.K., Khatib Y., Linklater J., Murrell G.A. (2007). Restore orthobiologic implant: Not recommended for augmentation of rotator cuff repairs. J. Bone Jt. Surg. Am..

[109] Zheng M.H., Chen J., Kirilak Y., Willers C., Xu J., Wood D. (2005). Porcine small intestine submucosa (SIS) is not an acellular collagenous matrix and contains porcine DNA: Possible implications in human implantation. J. Biomed. Mater. Res. Part B Appl. Biomater..

[110] Kokkalis Z.T., Mavrogenis A.F., Scarlat M., Christodoulou M., Vottis C., Papagelopoulos P.J., Sotereanos D.G. (2014). Human dermal allograft for massive rotator cuff tears. Orthopedics.

[111] Ahmed J., Varshney S.K. (2011). Polylactides—Chemistry, properties and green packaging technology: A review. Int. J. Food Prop..

[112] Chen J., Xu J., Wang A., Zheng M. (2009). Scaffolds for tendon and ligament repair: Review of the efficacy of commercial products. Expert Rev. Med. Dev..

[113] Longo U.G., Lamberti A., Khan W.S., Maffulli N., Denaro V. (2011). Synthetic augmentation for massive rotator cuff tears. Sports Med. Arthrosc. Rev..

[114] Gillespie R.J., Knapik D.M., Akkus O. (2016). Biologic and synthetic grafts in the reconstruction of large to massive rotator cuff tears. JAAOS-J. Am. Acad. Orthop. Surg..

[115] Emadi H., Karevan M., Masoudi Rad M., Sadeghzade S., Pahlevanzadeh F., Khodaei M., Khayatzadeh S., Lotfian S. (2023). Bioactive and biodegradable polycaprolactone-based nanocomposite for bone repair applications. Polymers.

[116] Chen C., Zhu J., Chen J., Yu F., Huang K., Jiang J., Zhu T., Mo X., Zhao J. (2022). A reinforced nanofibrous patch with biomimetic mechanical properties and chondroinductive effect for rotator cuff tissue engineering. Mater. Today Chem..

[117] Ozbolat I.T., Hospodiuk M. (2016). Current advances and future perspectives in extrusion-based bioprinting. Biomaterials.

[118] Mandalia K., Mousad A., Welborn B., Bono O., Le Breton S., MacAskill M., Forlizzi J., Ives K., Ross G., Shah S. (2023). Scaffold-and graft-based biological augmentation of rotator cuff repair: An updated systematic review and meta-analysis of preclinical and clinical studies for 2010–2022. J. Shoulder Elb. Surg..

[119] Ogueri K.S., Laurencin C.T. (2020). Nanofiber technology for regenerative engineering. ACS Nano.

[120] Qi Y., Niu L., Zhao T., Shi Z., Di T., Feng G., Li J., Huang Z. (2015). Combining mesenchymal stem cell sheets with platelet-rich plasma gel/calcium phosphate particles: A novel strategy to promote bone regeneration. Stem Cell Res. Ther..

